# Silver nitrate: a novel therapeutic approach for refractory Seroma following body malodor surgery

**DOI:** 10.1093/jscr/rjae067

**Published:** 2024-02-13

**Authors:** Wen-Tsao Ho

**Affiliations:** Department of Dermatology, Ho Wen Tsao Skin Clinic, New Taipei city 244, Taiwan

**Keywords:** seroma, silver nitrate, axillary osmidrosis, refractory

## Abstract

Seroma, a fluid collection that can develop after surgery, can be a challenging complication to manage. Conventional treatment options, such as quilting suture and drainage tubes, may not be effective in resolving refractory seromas. This article presents two cases of refractory seroma after axillary osmidrosis surgery that were successfully treated with silver nitrate. Silver nitrate, a topical agent with antiseptic, anti-inflammatory, and wound-healing properties, has been shown to be effective in treating perianal fistulas and persistent tracheocutaneous fistulas. In both cases presented here, silver nitrate resulted in complete seroma resolution within 7 and 14 days, respectively. This study suggests that silver nitrate may be a promising treatment option for refractory seroma after axillary osmidrosis surgery. Further research is warranted to validate these findings and establish optimal dosage and treatment protocols.

## Introduction

Osmidrosis, also known as body malodor, is a common condition characterized by the production of foul-smelling sweat. Surgery is the most effective treatment for osmidrosis, but it can be associated with complications, including hematoma and rarely, seroma [1]. Seroma is a fluid collection that can occur in the surgical wound. It is typically caused by disruption of the lymphatic drainage system. Seroma can usually resolve on its own within a few weeks, but larger or persistent seromas may require treatment.

Treatments for seroma include quilting suture, drainage, sclerotherapy, and surgical excision. Quilting suture is a simple and effective method, but it may not be sufficient for larger seromas. Drainage can be effective, but it can be uncomfortable and may increase the risk of infection. This study reports two cases of refractory seroma after osmidrosis surgery that were successfully treated with silver nitrate. These cases suggest that silver nitrate may be a useful treatment option for seroma after osmidrosis surgery.

### Case report 1

**Figure 1:**
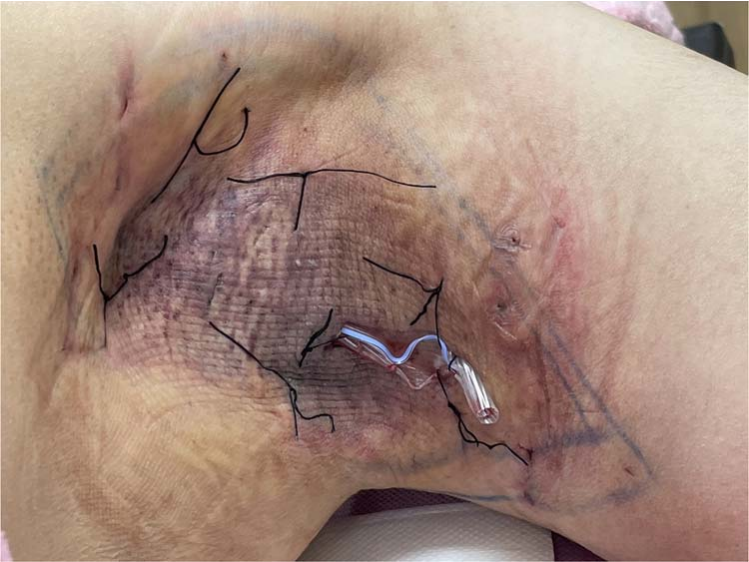
The seroma was treated with quilting suture and Penrose drain placement. The quilting suture approximated the skin flap to the underlying tissues to reduce dead space and promote fluid reabsorption, while the Penrose drain directly drained accumulated fluid from the seroma cavity.

A 24-year-old female patient underwent surgery for body odor. The healing process was uneventful, but one month postoperatively, she presented with a soft, 6 cm x 3 cm mass in her left axilla. Following local anesthesia, a small incision was made over the mass, and a clear exudate was drained. The seroma was then treated with quilting suture and Penrose drain placement (Fig. 1). After two weeks of observation, the skin flap remained easily separable from the subcutaneous tissue. Based on our literature review, we found that silver nitrate has been used successfully to treat perianal fistula and persistent tracheocutaneous fistulas [2, 3]. Therefore, we decided to try 20% silver nitrate to treat the patient’s seroma to the seroma cavity. The patient experienced immediate pain at the application site, which subsided completely the next day. The seroma continued to decrease in size over the next few days, and it was completely resolved within 7 days.

### Case report 2

**Figure 2:**
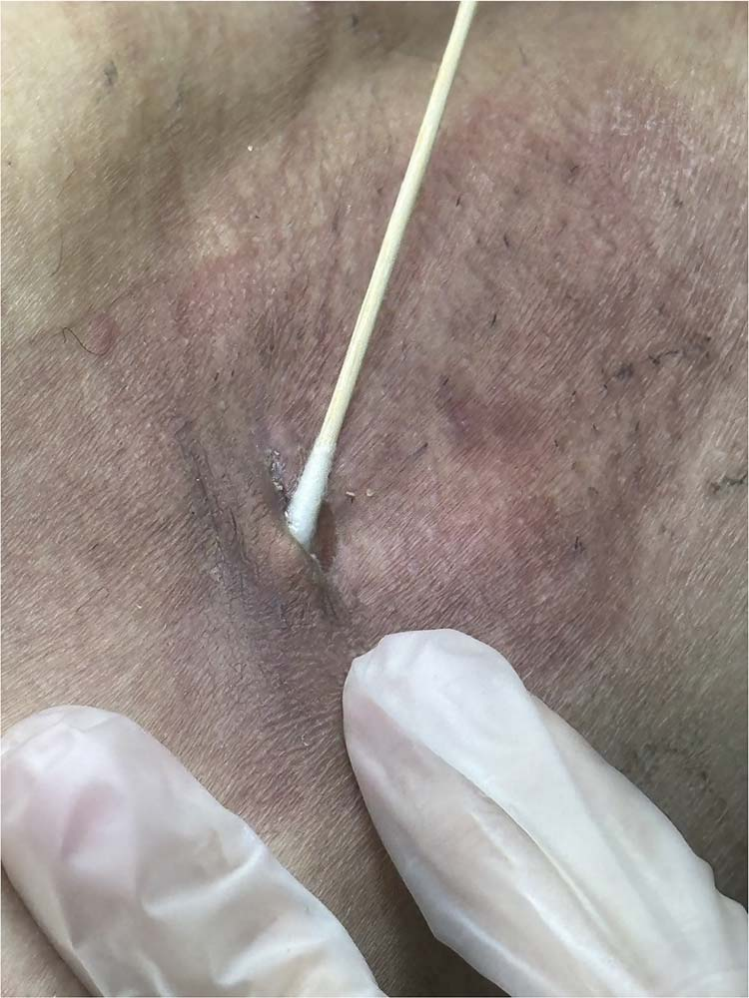
The seroma responded to silver nitrate treatment. A second application of silver nitrate, applied using ENT cotton swab, resulted in complete seroma resolution within 14 days. This figure demonstrates the efficacy of silver nitrate in treating seroma.

A 21-year-old male patient presented with a 3 cm x 3 cm seroma in his left axilla. The seroma was initially treated with a Penrose drain, which was removed after one week because the seroma had dried up. However, the seroma recurred two weeks later. The Penrose drain was reinserted, but the seroma recurred again after two weeks. This cycle repeated three times. We applied a 20% silver nitrate solution to the seroma cavity. The patient experienced immediate pain at the application site, which subsided within a few hours. The seroma continued to decrease in size in the next week and we reapplied silver nitrate again (Fig. 2). It was completely resolved within 14 days.

In both cases, silver nitrate was effective in resolving seroma after surgery for body odor. Patients may experience a significant amount of clear exudate from the seroma cavity for the first few days after silver nitrate treatment. This is likely due to the irritation caused by the silver nitrate. The exudate typically decreases significantly within three days. Both patients were followed for six months after treatment, and no recurrence of seroma was observed.

## Discussion

Currently, most of the literature on seroma is related to breast cancer. According to one study, the risk of seroma after breast cancer surgery is approximately 12.8% [4]. The literature on osmidrosis surgery mentions seroma is 1.2% [5]. In my experience, the incidence of seroma after osmidrosis surgery is extremely rare.

Conventional treatment options for seroma, such as quilting suture and drainage tubes, proved ineffective in resolving the seromas in our patients. Quilting suture involves approximating the skin flap to the underlying tissues to reduce dead space and promote fluid reabsorption, but it is particularly effective for small seromas located close to the skin surface [6]. Drainage tubes, such as Penrose drains, are inserted into the seroma cavity to directly drain accumulated fluid, making them suitable for larger seromas and providing immediate relief from fluid pressure [7]. Sclerotherapy involves injecting sclerosing agents, such as tetracycline, into the seroma cavity, inducing inflammation and fibrosis within the seroma space to cause its collapse and obliteration [8–10]. However, our clinic lacks access to these medications, limiting their use as treatment options.

A comprehensive literature review on the successful use of silver nitrate in fistula treatment inspired us to consider its application for seroma. Silver nitrate is a strong antiseptic with anti-inflammatory and wound-healing properties, making it a promising candidate for seroma management [11].

This innovative approach yielded remarkable results. Both patients experienced complete seroma resolution following silver nitrate treatment. Our case series provides compelling evidence supporting the potential efficacy of silver nitrate in treating seroma, particularly in cases where conventional treatments have failed. Further research is warranted to validate these findings and establish optimal dosage and treatment protocols.

## Conclusion

This study presents two cases of refractory seroma after axillary osmidrosis surgery that were successfully treated with silver nitrate. These findings suggest that silver nitrate may be a promising treatment option for refractory seroma after axillary osmidrosis surgery. Further research is warranted to validate these findings and establish optimal dosage and treatment protocols.
